# Short Trajectory Segmentation With 1D Unet Framework: Application To Secretory Vesicle Dynamics

**DOI:** 10.1109/ISBI45749.2020.9098426

**Published:** 2020-05-22

**Authors:** Mariia Dmitrieva, Joël Lefebvre, Kristofer delas Peñas, Helen L Zenner, Jennifer Richens, Daniel St Johnston, Jens Rittscher

**Affiliations:** 1 Department of Engineering Science, University of Oxford, Oxford, UK; 2 Gurdon Institute, University of Cambridge, Cambridge, UK; 3 Department of Computer Science, University of the Philippines, Quezon City, Philippines

**Keywords:** protein trafficking, trajectory segmentation, deep learning, U-Net, sliding window

## Abstract

The study of protein transport in living cell requires automated techniques to capture and quantify dynamics of the protein packaged into secretory vesicles. The movement of the vesicles is not consistent along the trajectory, therefore the quantitative study of their dynamics requires trajectories segmentation. This paper explores quantification of such vesicle dynamics and introduces a novel 1D U-Net based trajectory segmentation. Unlike existing mean squared displacement based methods, our proposed framework is not restricted under the requirement of long trajectories for effective segmentation. Moreover, as our approach provides segmentation within each sliding window, it enables effectively capture even short segments. The approach is quantified by the data acquired from spinning disk microscopy imaging of protein trafficking in *Drosophila* epithelial cells. The extracted trajectories have lengths ranging from 5 (short tracks) to 135 (long tracks) points. The proposed approach achieves 77.7% accuracy for the trajectory segmentation.

## 1 Introduction

Advances in live-cell imaging provide us with the opportunity to image diverse biological processes and organelle structures within living cells at a sub-micron resolution. Dynamics of the secretory vesicles contains information about their interaction with the complex environment and provides a key for understanding the mechanism of protein transportation within living cells. Therefore advancing these studies hinges on particle tracking and quantitative analysis of the secretory vesicles dynamics.

Various tracking algorithms have been developed in recent years [1]. The single particle tracking techniques determine trajectories of the labelled vesicles. However, further analysis of these trajectories is required to study vesicles dynamics. A variety of factors govern vesicle movement which is why a single trajectory can contain different motion types. Temporal segmentation and classification are one approach for identifying changes in motion. In this paper, given computed trajectories, we explore the quantification of vesicle dynamics via their trajectory segmentation.

A number of groups utilise mean squared displacement (MSD) to address the task of trajectory segmentation. While MSD provides a high accuracy level, it requires long trajectories to analyse the particle dynamics. Liu *et al*. in [2] exploited sliding window along the trajectory, while calculating MSD curve for each segment with a segment size of 80 points. The trajectory segments were classified into directed diffusion, Brownian diffusion, confined diffusion and immobilisation. Hue and collaborators [3] evaluated the particle motion based on the diffusion coefficient, MSD curvature and trajectory asymmetry with sliding window along the trajectory. It required a minimum window length of 11 points for asymmetry, and 20 points for the MSD and diffusion coefficients evaluation to discriminate between stalled vesicles, diffusive class, directed class and constrained class. Yin *et al*. [4] considered changes in the diffusion coefficient and velocity with a trajectory length from 20 to 1000 points. The approach classifies trajectory’s segments into two types: directional movement and random walk. Monnier *et al*. in [5] proposed a Bayesian approach to compute probabilities of an arbitrary set of motion models taking into account MSD, which leads to the 40 points trajectory length.

A nonparametric three-decision test [6] was proposed as an alternative to the MSD-based methods. Here trajectories are classified into free diffusion (Brownian motion), subdiffusion (including confined motion) and superdiffusion with a minimum sliding window length of 10 points. Wagner *et al*. in [7] presented random forest based classification with 9 different features. The original trajectory (40 points and above) is divided into overlapping segments and classified into four basic motion types: normal diffusion, anomalous diffusion, confined diffusion or directed motion. Garnik *et al*. [8] presented deep learning based approach classifying trajectories by diffusion type: Brownian motion, fractional Brownian motion and continuous time random walk. The approach requires trajectory length of 25 points.

The existing trajectory segmentation approaches are limited by the minimum track length, as they require long trajectories for the accurate dynamics analysis. Therefore, short trajectories with a length less than 10 points cannot be segmented or classified by the existing solutions. In this paper, we propose a novel trajectory segmentation approach which doesn’t require long tracks. The approach is based on the U-Net architecture [9]. The U-Net is a common solution for 2D or 3D image semantic segmentation and recently 1D U-Net was exploited for the audio source separation [10]. In this work, we adapt the U-Net architecture to the trajectory segmentation. The sliding window along the trajectory is exploited and each of the sliding sections is segmented separately, then all the sections are combined to the final segmentation result. The minimum segment size is not limited by the window length as a single window can contain different classes. In this way, the sliding window approach is different from the methods where each window is classified into a motion type (e.g. [2], [3], [5]), limiting the minimum segment size to the window length.

The proposed approach segments directed motion, which also can be described as superdiffusion, from the rest of the trajectory. Therefore the trajectories are segmented into two classes: *moving* (the directed motion) and *not-moving* class. The *not-moving* class can be characterised by stalled behaviour, Brownian and confined motion. The contribution of this work is the adaptation of the U-Net architecture to the trajectory segmentation task, a proposed approach for the trajectory normalisation and a comparison of the segmentation results obtained for different coordinate systems.

## 2 Method

**Fig. 1:**
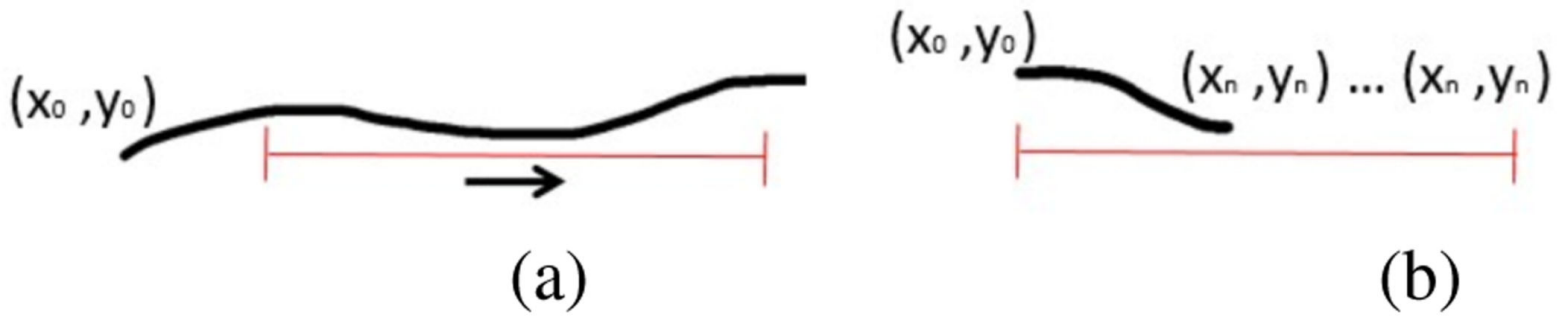
Example of the sliding window (red) over a trajectory (black curve): (a) with a trajectory length above the window size; (b) with a trajectory length less than the window size.

Our trajectory segmentation approach is based on the U-Net architecture with its contracting (down-sampling) and expanding (up-sampling) paths. Sliding windows are used to move along trajectories with a window size *W* = 8 points, where each point is equivalent to one sequential frame. The sliding step of the window is equal to 25 % of its length and only the centre part (50 %) is incorporated into the final segmentation for all segments apart from the first and the last window. The proposed parameters of the sliding window are chosen empirically. The length of the trajectories can vary from a few to more than a hundred frames. In case the trajectory length is less than *W* a padding with constant values is applied (see Fig. 1). This duplication is considered as a stalled behaviour and associated with the not-moving class.

### 2.1 Trajectory pre-processing

Each partition of the trajectory (a single sliding window) has to be normalised before passing it to the 1D U-Net for the segmentation. The process is different from the image normalisation as the trajectories are normalised with respect to their first position:

(1) $$T_{\text{norm}\;}(x,y)=T(x,y)-\left(x_{0},y_{0}\right),$$

where *T* (*x, y*) is a trajectory within one sliding window and (*x*_0_, *y*_0_) is the first coordinates of the section. It translates all the segments to the same origin, while preserving proportion of their displacement which is essential for the dynamic evaluation inside of each segment.

**Fig. 2:**
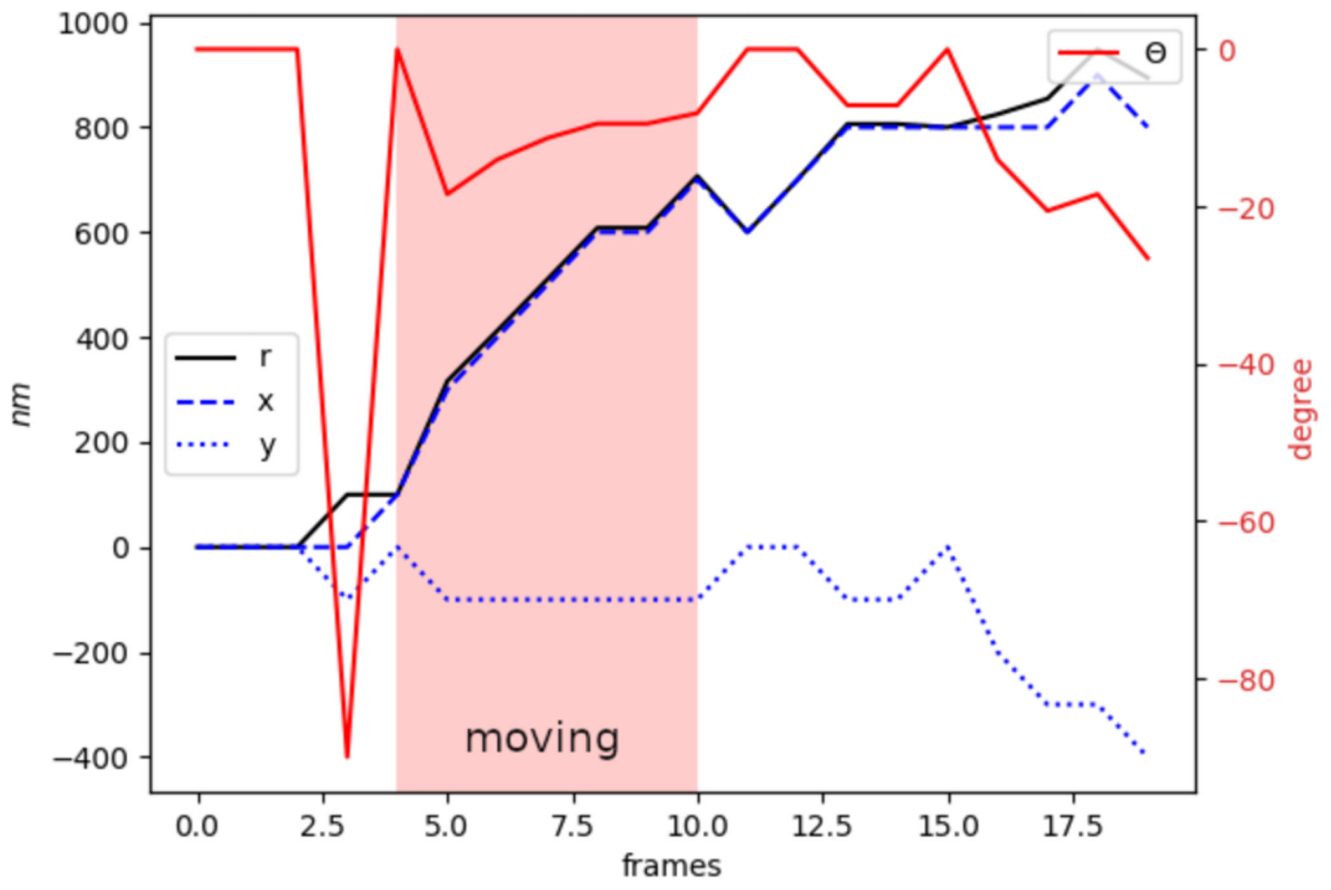
Comparison of the trajectory representation in Cartesian (*x, y*) and polar (*r, θ*) coordinates after normalisation.

Another important aspect of data pre-processing is the choice of the coordinate system. The original trajectories are presented in the Cartesian coordinate system, while empirically we found polar coordinates more efficient for the network training. The distance to the reference point (*r*) in polar coordinates represents displacement by a single value, while Cartesian system represents the same data in more complex way with *x* and *y* coordinates which are not always following the same trend. Fig. 2 illustrates it with an example of a trajectory where the vesicle is moving in *x* direction. The major changes in the position for Cartesian coordinates is represented by *x*, while *y* doesn’t change much, which would be opposite for the case of vesicle movement in *y* direction.

Contrarily, in polar coordinates *r* always represents the displacement and therefore can be a better choice for the network training. In this work we converted trajectory to the polar coordinates:

(2) $$r=\sqrt{x^{2}+y^{2}}\;\;\text{and}\;\;\theta ={\text{tan}}^{-1}\left(\frac{y}{x}\right),$$

where (*x, y*) are Cartesian coordinates and (*r, θ*) are polar coordinates. In this way, each sliding window section is normalised, converted to the polar coordinate system and passed to the 1D U-Net for further segmentation.

### 2.2 1D U-Net

**Fig. 3:**
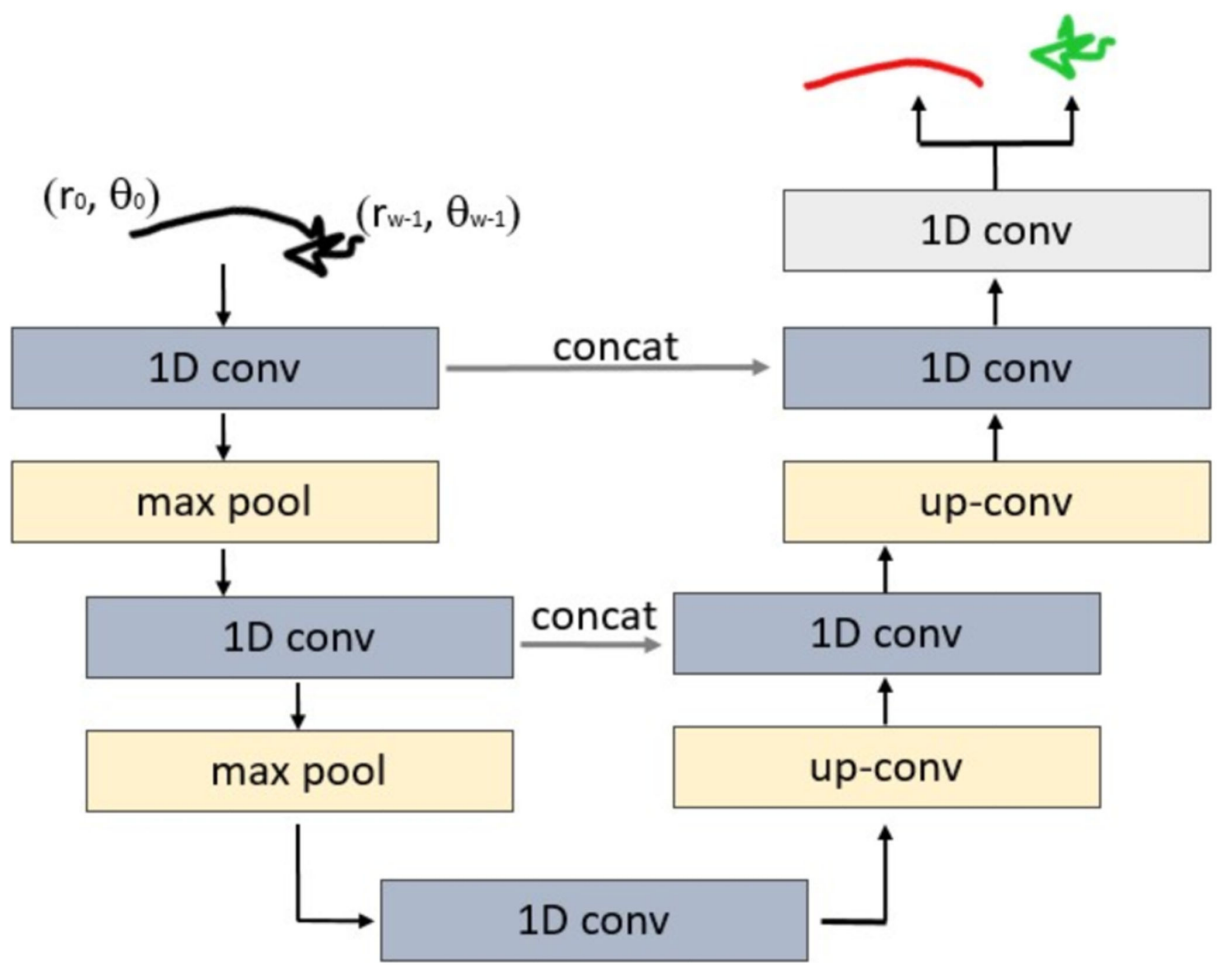
Proposed 1D U-Net architecture for two class segmentation: *moving* (red) and *not-moving* (green) segments.

A diagram of the proposed 1D U-Net architecture is presented in Fig. 3. The network consists of 2 down-sampling and 2 up-sampling blocks connected by skip connections. A single down-sampling block contains one 1D convolution (1D conv) and a max pooling (max pool). In the up-sampling blocks the pooling operation is replaced by the up-sampling operator (up-conv). Each 1D convolutional layer is followed by a rectified linear unit (ReLU). The down-sampling is implemented by the max pooling operation with stride 2. The final layer consists of a 1D convolution followed by a sigmoid activation. It maps each feature vector to the two classes. The network is trained with the Adam optimizer with learning rate of 0.0001. For minimisation criteria we use the cross entropy loss function.

## 3 Results

The proposed approach is evaluated on the data obtained by the spinning disk microscopy of protein trafficking in epithelial cells of the *Drosophila* egg chamber. The current experimental data contains two channels, temporal resolution is set to 4 *f/s* with spatial resolution of 100 *nm/pixel*. The labelled protein can be detected throughout its passage in the secretory system, in the Golgi, in vesicles and at the plasma membrane.

As our imaging protocol only captures a single z-slice with a limited depth of field, while vesicles have 3D trajectories and can be traveling in z-direction, we obtain a very large number of short trajectories. The trajectories are computed from the original video sequences by the tracking algorithm described in [11]. Overall 5 video sequences, each within a single cell are processed by the tracker. The results were reviewed manually and 181 trajectories were selected to compose a dataset. Each trajectory in the dataset was manually annotated based on the visual observation of the vesicle movements. The network was trained on the trajectories from 4 video sequences (140 trajectories) and tested on 1 remaining sequence (41 trajectories). As a sliding window was used for the trajectory segmentation, in total the training dataset consist of 2066 samples.

The results of the approach are evaluated by the accuracy of the segmentation. The first point in each trajectory is not taken into account for the evaluation, as the first position by itself cannot represent the vesicle dynamics. The accuracy of the approach is quantified on the testing partition of the dataset and the proposed approach achieves 77.7% accuracy while exploiting polar coordinates and 8 points sliding window length.

The 1D U-Net was trained with a trajectories expressed in the polar coordinates. Tab. 1 presents a comparison of the results for training on the same data represented in different coordinate systems. Although the accuracy improvement is small, the table shows that the choice of the coordinate system affects the segmentation results. Our data indicates that the polar coordinate system provides higher accuracy.

**Fig. 5:**
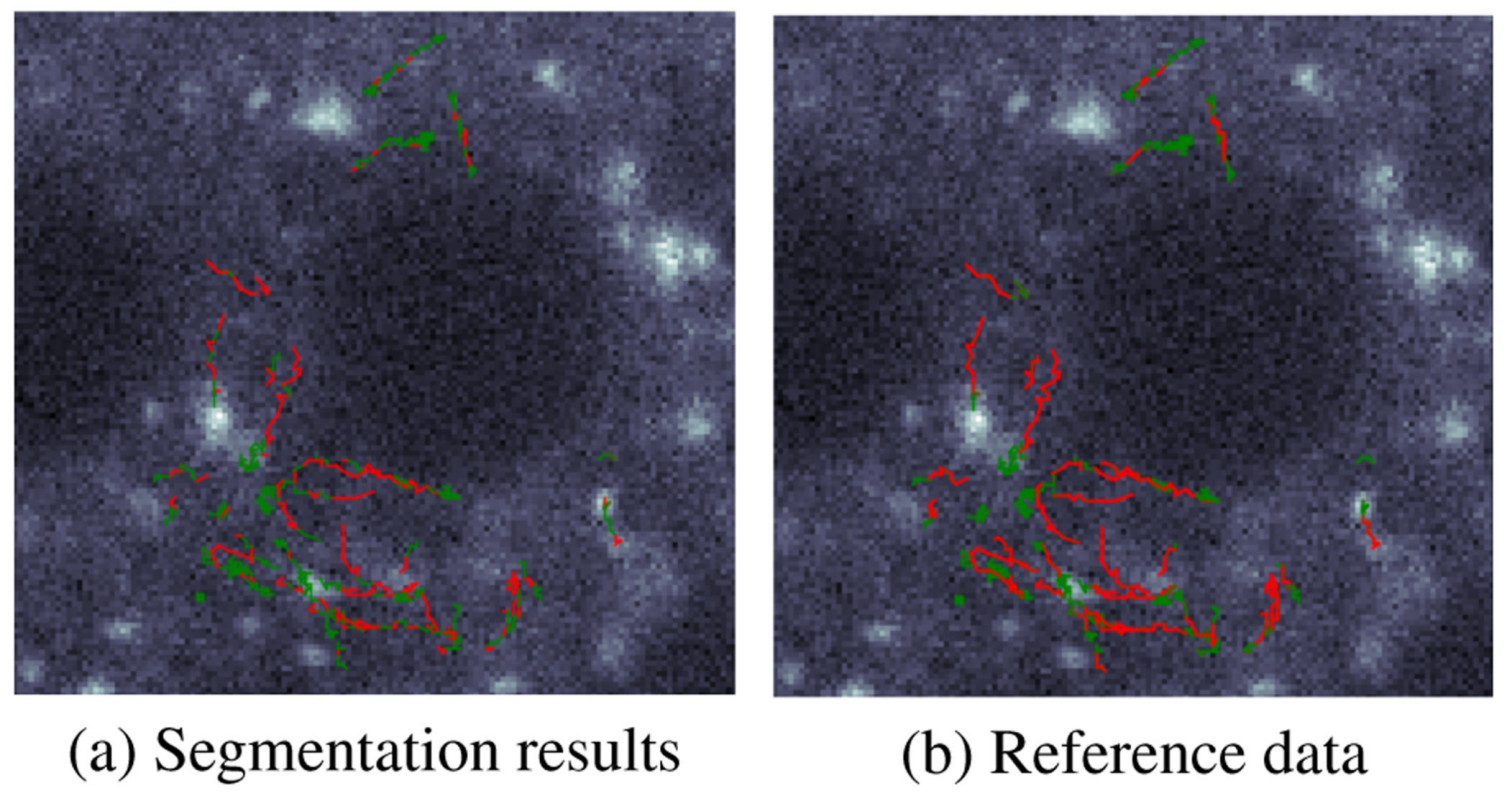
Visual quantification of the segmentation results (a) in comparison with manually annotated reference data (b): red colour - *moving* segments, green - *not-moving* segments.

The visual quantification of the segmentation results in comparison with manually annotated reference data is presented in Fig. 5. Colour of the trajectories represents the segmentation class with red colour for the directed motion and green colour for the remaining trajectories. It can be seen that the directed movement is well captured by the approach, while some of the confined and Brownian motion segments are misclassified as a directed motion.

## 4 Conclusions

This paper introduces a novel approach to trajectory segmentation. Unlike existing methods, the proposed approach can handle short trajectories. This framework introduces a 1D U-Net architecture and a sliding window based approach. For each sliding window an independent segments are computed and therefore the smallest segment size is not limited by the window length. Thus, the sliding window approach has an advantage over window based solutions where entire window is classified into a movement type thereby limiting the minimum segment size to the window length.

The proposed approach is evaluated on the original data of secretory vesicle trafficking in epithelial cells of the *Drosophila* egg chamber for which the smallest trajectory length is 5 points. This approach is not compared with solutions described in Section 1 as longer trajectory lengths are required for trajectory segmentation with these methods and therefore many trajectories from the original data cannot be processed.

In future work, this segmentation approach can be extended to a multiple classes by separating different motion types within existing class. For instance, stalled and constrained motion can be segmented within the *not-moving* class. This will require an enlargement of the dataset and accurate manual data annotation. The approach can be extended to the 3D movement, which would require a training 3D data. Furthermore, the proposed approach can be adapted for the orientation based trajectory segmentation. It would allow to study orientation of the vesicle trajectories and will be beneficial for the biological studies of the protein trafficking within living cells.

## Figures and Tables

**Fig. 4:**
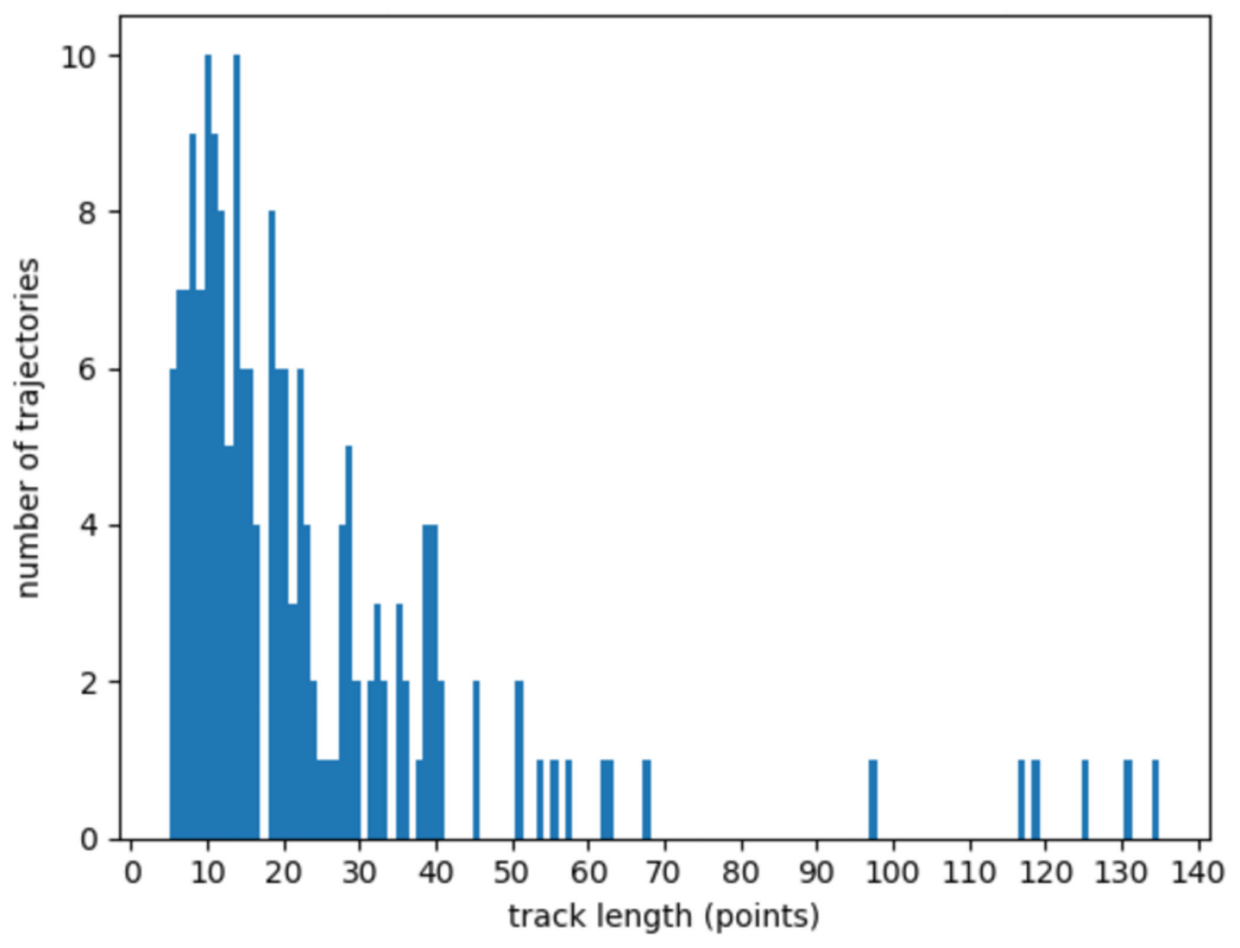
Experimental dataset: histogram of the trajectories length. The data contains a large percentage of tracks which could not be analysed using existing methods.

**Table 1:** Experimental results of the proposed framework exploiting different coordinate systems.

Coordinate system	accuracy
Cartesian	76.8%
Polar	**77.7%**
